# Molecular Composition and Antibacterial Effect of Five Essential Oils Extracted from *Nigella sativa* L. Seeds against Multidrug-Resistant Bacteria: A Comparative Study

**DOI:** 10.1155/2021/6643765

**Published:** 2021-03-15

**Authors:** Mohammed Dalli, Salah-eddine Azizi, Hind Benouda, Ali Azghar, Maroua Tahri, Boufalja Bouammali, Adil Maleb, Nadia Gseyra

**Affiliations:** ^1^Laboratoire de Bioressources, Biotechnologie, Ethnopharmacologie et Santé, Equipe de Physiologie et Ethnopharmacologie, Université Mohammed Premier, Faculté des Sciences, Bloc de Recherche 1ème étage 60 000, Oujda, Morocco; ^2^Laboratoire de Chimie Organique, Macromoléculaire et Produits Naturels, Université Mohammed Premier, Faculté des Sciences, Bloc de Recherche 2ème étage 60 000, Oujda, Morocco; ^3^Laboratoire de Microbiologie, Centre Hospitalier Universitaire (CHU), Oujda, Morocco

## Abstract

*Nigella sativa* L. *(NS)* and its volatile compounds are well known for their broad spectrum of effects. This study aimed to investigate the variability of the chemical composition and the *in vitro* antibacterial activity of five essential oils (Eos) originated from Morocco, Saudi Arabia, Syria, India, and France. These five samples were grown under different edaphic and climatic conditions. The agar diffusion method and microdilution method in 96-well plates were used to test the sensitivity of multidrug-resistant strains clinically isolated from patients (methicillin-resistant *Staphylococcus aureus*, *Escherichia coli*, *Pseudomonas aeruginosa*, and *Acinetobacter baumannii*), for the determination of the minimum inhibitory concentration and bactericidal concentration. Among all the investigated Eos, the monoterpenes were highly present in the chemical composition. Moroccan, Saudi Arabian, and Syrian seeds were characterized by the presence *α*-phellandrene (20.03–30.54%), *β*-cymene (12.31–23.82 %), and 4−caranol (9.77–14.27%). The Indian seeds were rich with 4-caranol (18.81%), *β*-cymene (14.22%), *α*-phellandrene (10.58%), and *β*-chamigrene (9.54%), while France *NS* was rich with estragole (20.22%) and D-limonene (14.63%). The minimum inhibitory (MIC) and bactericidal concentration (MBC) obtained for the four Eos (with the exception of France because of the low yield) tested were ranging from 3 to 40 *μ*l/ml. Gram-positive (+) bacteria were slightly sensitive to the Eos tested than the Gram-negative (−) bacteria. The results of this study showed that the Eos of *NS* seeds show interesting antibacterial activity which could be associated to the existence of different bioactive compounds. Indeed, these compounds can be used for preventive or curative purposes in the face of the noncontrolled emergence of resistance to antibiotics.

## 1. Introduction

Multidrug resistance to antibiotics has become a serious problem that threatens millions of people around the world. This resistance has led to an increase in research for new alternatives such as medicinal plants [1]. For this fact, the World Health Organization (WHO) has developed a list of global priority pathogens of multidrug-resistant bacteria to develop new effective antibiotic treatments [2].

Essential oils (Eos) are also named volatile compounds of the plant and known for their various benefits such as antibacterial, antifungal, and antiviral properties. The Eos are considered a major source in different domains such as perfumeries, cosmetics, and the pharmaceutical industry [3].

*Nigella sativa* L. *(NS)* (black cumin) annual herbaceous belonging to the Ranunculaceae family was largely used in folk medicine for centuries [4]. The plant is widely distributed in North Africa, the Middle East, and India. It is used for the treatment of various illnesses such as cough, asthma, fever, and eczema and for its benefit as a lactogogue and emmenagogue [5]. Nowadays, different biological studies are being established on *NS*; the richness of their seeds with volatile compounds, saponins, and alkaloids [6] provide a large spectrum of effects on different diseases [7]. Recently, it was proved that *NS* oil and extracts exert an antimicrobial, immunomodulatory, and anticancer activity [8].

The objective of this research is to study the chemical variation of the different *NS* Eos originated from India, Saudi Arabia, Morocco, Syria, and France and to evaluate their potential on some of the most common pathogenic multidrug-resistant bacteria: methicillin-resistant *Staphylococcus aureus*, *Escherichia coli*, *Pseudomonas aeruginosa*, and *Acinetobacter baumannii* isolated from the hospital.

## 2. Materials and Methods

### 2.1. Materials

#### 2.1.1. *Nigella sativa* Seeds

The studied *Nigella sativa* seeds originate from five countries: Saudi Arabia, India, Syria, France, and Morocco. The determination of the scientific name of plant species was performed, verified, and confirmed with a taxonomist/professional botanist using live specimens and photographs. For the accuracy of the identification of plant species, we have referred to specialized documents related to Moroccan flora. A specimen was deposited at the faculty herbarium under the voucher number **(HUMPOM471)** [9–14].

#### 2.1.2. Bacterial Strains

The bacteria used for the determination of the antibacterial activity (*S.aureus*, *E.coli*, *P.aeruginosa,* and *A.baumannii*) were clinically isolated from patients and identified at the microbiology laboratory of the University Hospital Centre Mohammed VI of Oujda (Morocco). Their sensitivity toward antibiotics was verified according to the European Committee on Antimicrobial Susceptibility Testing (EUCAST). The chosen strains are representative of the most commonly involved pathogen-resistant bacteria in human infections.

#### 2.1.3. Antimicrobial Susceptibility Testing

The *in vitro* antibiotic susceptibility test based on the disk diffusion method was used in this study. The isolated strains' sensitivity was accessed according to the EUCAST. The antibiotics tested and their concentrations are cefoxitin 30 *μ*g for methicillin-resistant *S. aureus*, imipenem 10 *μ*g for *Acinetobacter* resistant to carbapenem, and ceftazidime 10 *μ*g for the ceftazidime-resistant *Pseudomonas*. For extended-spectrum beta-lactamases (ESBL), amoxicillin clavulanic acid 30 *μ*g, cephalosporins third generation (Cefotaxime 5 *μ*g) were used.

#### 2.1.4. Chemicals

The following reagents and solvents were purchased from Sigma-Aldrich chemicals: hexane, dimethylsulfoxide (DMSO), and rizasurine sodium salt.

### 2.2. Essential Oil Extraction

The *NS* samples were placed in the shady room to be naturally dried until their weight was stable for (6 days) approximately. The fully dried samples were turned to a fine powder before hydrodistillation. Then, 100 g of plant material in 300 ml of water were subjected to hydrodistillation using a modified Clevenger-type apparatus until the essential oil level was stable (2 to 3 h). After the extraction, anhydrous sodium sulfate was used to remove water trace, and the essential oil was stored in an airtight glass container in a refrigerator at 4°C until the analysis.

### 2.3. Qualitative and Semiquantitative Analysis of *Nigella sativa* Essential Oil

The analysis was performed using a gas chromatograph (Shimadzu GC-2010) equipped with a fused-silica capillary column (5% phenyl methyl siloxane, 30 m × 0.25 mm, 0.25 *μ*m film thickness) coupled with a mass spectrometer detector (GC-MS-QP2010). The helium as a carrier gas was adjusted to a constant pressure of 100 KPa. The oven temperature was set initially at 50°C (maintained for 1 minute) followed by a gradient of 10°C/min up to 250°C (maintained for 1 minute). The temperatures of the injector, transfer line, and ion source were set at 250°C, 250°C, and 200°C, respectively. For the qualitative and semiqualitative analysis (Table 1), solutions containing 1 *μ*L of the samples diluted in hexane (50 mg/g) were injected in split mode (split ratio = 50–80), and the GC-MS system was operated in scan mode. Mass spectra were recorded at 70 eV (electron impact ionization mode) with an m/z range of 40–350 a.m.u (rate and solvent delays were 5 s/scan and 4.5 minutes, respectively). Identification of the essential oil constituents was accomplished based on the comparison of their MS data with those stored in the National Institute of Standards and Technology (NIST147) computer library. LabSolutions (version 2.5) was used for data collection and processing. Table 1 gives the relative percentage of each component of the studied Eos according to their GC peak areas without correction factors. All experiments were carried out in triplicate, and data was expressed as the mean ± SD.

### 2.4. Determination of the Antibacterial Activity

The antibacterial activity of *NS* Eos has been determined using two different techniques as described below.

#### 2.4.1. Agar Diffusion Method

The agar diffusion method was used as a preliminary test for the determination of the Eo antibacterial activity. The turbidity of all strains was standardized to 0.5 McFarland to facilitate the comparison between them. Afterward, the strains were inoculated on the surface of a Petri dish of Muller Hinton Agar (MHA) using a sterilized swab. Sterilized Whatman filters (6 mm in diameter) were saturated with 20 *μ*l of Eo dissolved in 2% DMSO, and then, they were placed on the surface of the Petri dish previously inoculated with microorganisms using forceps. After 24 hours of incubation at 37°C of all the dishes, the antibacterial activity was assessed by measuring the diameter of the growth-inhibition zone in millimeter. The measurements of inhibition zones were carried out three times for each Eo [15].

#### 2.4.2. Determination of Minimal Inhibitory (MIC) and Bactericidal (MBC) Concentrations

In this study, the microdilution method using a 96-well plate was adopted. Resazurin was utilized as an indicator of bacterial growth for the determination of MIC [16]. Resazurin is a nonfluorescent purple/blue dye that becomes pink and fluorescent when reduced to resorufin by oxidoreductase enzymes in viable cells [17].

The essential oil was dissolved in 2% DMSO, and a serial dilution was prepared in Muller Hinton broth. After the stirring, 180 *μ*l of each concentration was added to the plate wells. Then, a suspension of bacteria cells (10^7^ CFU/ml, 20 *μ*l) was added to each well. After adding resazurin, all the plates were incubated at 37°C for 24 h. The smallest concentration of essential oil that shows no color variation was considered as the MIC.

Afterward, 20 *μ*l is withdrawn from each well of the microplate, which does not show any color change, spread on the surface of the MHA Petri dish using a sterilized swab, and then, incubated at 37°C for 18 to 24 hours. The dish with no subculture was considered as the MBC.

All tests were performed in triplicates including the antibiotic MHB as a positive control and essential oil dissolved in DMSO 2% without the microorganism as a negative control.

### 2.5. Statistical Analysis

The analysis was performed with ANOVA followed by the Tukey test with post hoc multiple comparisons threshold 5%. The oil components with a percentage higher than 5% of the total oil were subjected to hierarchical cluster analysis (HCA) and principal component analysis (PCA) using SPSS v22.0 software. In the case of HCA, the dendrogram (tree) was produced using Ward's method of hierarchical clustering with the squared Euclidean distance between oil samples.

## 3. Results and Discussion

### 3.1. Extraction and Chemical Composition of Essential Oils

**Figure 1:**
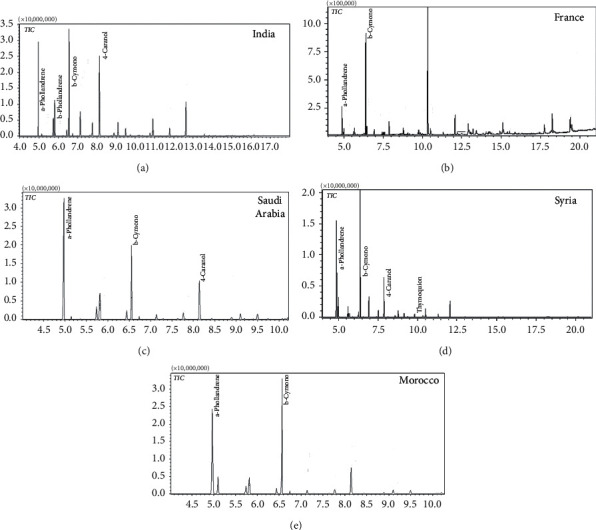
GC-MS Total Ion Chromatogram (TIC) of the *Nigella sativa* essential oils of five countries.

The essential oils (Eos) of five *NS* samples belonging to five countries (India, Saudi Arabia, Morocco, Syria, and France) were obtained by the hydrodistillation method in a yield ranging from 0.047% to 1.7%. The composition of the Eos was investigated using the GC-MS technique, and the identification of their constituents was accomplished based on the comparison of their MS data with those stored in the National Institute of Standards and Technology (NIST147) computer library. The chemical components and the chromatograms of the studied oils are shown in Table 1 and Figure 1, respectively. Of all of the identified compounds, monoterpenes constituted the highest percentage (55.81 to 96.68%). The data show that the major components were found to be *α*-phellandrene, *β*-pinene, *β*-cymene, and 4-caranol, respectively, for *NS* collected from India, Saudi Arabia, Morocco, and Syria. Oil compositions of France were characterized by the occurrence, at appreciable content, of estragole and D-limonene.

### 3.2. Chemical Variation in Essential Oil Composition

The oil components with percentages higher than or equal 5% of the total oil were subjected to hierarchical cluster (HCA) and principal component analysis (PCA) in order to investigate the similarity and relationship between Eos composition of *NS* samples.

**Figure 2:**
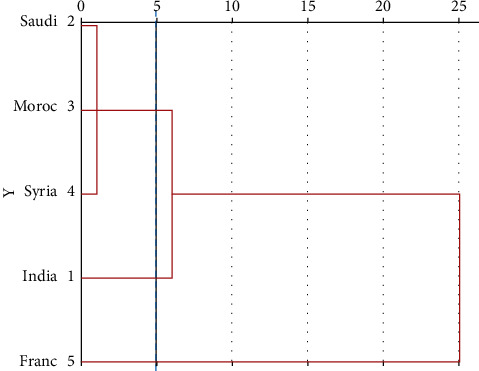
Dendrogram of five *Nigella sativa* populations produced by the hierarchical cluster analysis.

From a dendrogram produced by HCA (Figure 2), the *NS* populations can be classified into two main clusters at a distance of 25 units. Samples connected by a shorter distance are more similar than those connected by a longer distance. In the closer distances (∼5 units), the examined populations were divided into three groups (Gr 1 to 3). The first group (cluster I), represented by three samples originating from Saudi Arabia, Morocco, and Syria, was rich in *α*-phellandrene (20.03–30.54%) and had a relatively moderate level of *β*-cymene (12.31–23.82 %) and 4-caranol (9.77–14.27%). The second group, containing the sample of India and which is furthest from the other populations of cluster I, is characterized by the highest percentage of *β*-chamigrene (9.54%).

In the third group (cluster II), the France sample which constitutes the furthest group compared to the others had high and moderate levels in estragole (20.22%) and D-limonene (14.63%) respectively.

**Figure 3:**
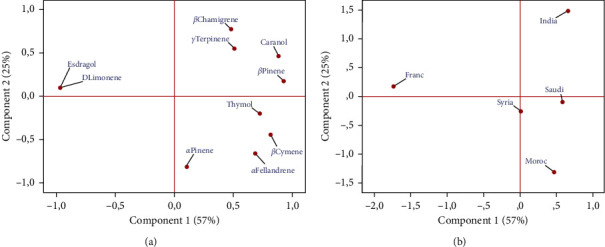
2D graphical representation of principal component analysis of chemical compositions oils of NS originated from five countries. (a, b) PCA distributions of variables and samples, respectively.

PCA analysis revealed that the first two principal components represented 82% of the phytochemical variance.  Figure 3 shows the 2D graphical representation of PCA. As shown in the figure, the first principal component (PC1) accounted for 57% of the total variance, correlated positively with *α*-phellandrene, *β*-pinene, *β*-cymene, and 4-caranol which were predominant in oils of *NS* collected from Saudi Arabia, Morocco, Syria, and India. On the other hand, PC1 correlated negatively with D-limonene and estragole which were predominant in oils of *NS* originated from France. The second principal component, accounting for an additional 25% of the total variance, was positively correlated with *β*-chamigrene. These PCA data lead to the classification of the *NS* into three main groups which represent the following chemotypes: *α*-phellandrene/*β*-cymene/4-caranol (Saudi Arabia, Morocco, and Syria), 4-caranol/*β*-cymene/*α*-phellandrene/*β*-chamigrene (India), and estragole/D-limonene (France). This classification confirmed the HCA results.

Finally, it is interesting to note the great similarity between Eos of *NS* samples collected from India, Saudi Arabia, Morocco, and Syria despite that they grew under different edaphic and climatic conditions. Such a close similarity of oil composition in plants can be explained by the similarity of the genetic factors.

### 3.3. Determination of the Antibacterial Activity

The sensitivity test showed that the different strains were resistant towards the used antibiotics, and no inhibition diameter was observed on the agar plate. Concerning the ESBL, the double-disc synergy method in Mueller Hinton agar was used. The amoxicillin clavulanic acid inhibits the production of beta-lactamases which create a synergic effect with cephalosporins third generation which highly suggest the production of ESBL.

*NS* Eos were evaluated for their antimicrobial activity against four bacteria-resistant strains*: S. aureus*, *E. coli*, *P. aeruginosa,* and *A. baumannii*. The data pertaining to the antimicrobial potential of *NS* Eos are presented in Table 2.

Among the tested bacteria, the multiresistant *S. aureus* (MRSA) was considered as sensitive to all Eos tested (zone inhibition: 14 to 15 mm) except for the Saudi Arabian Eo which gave a moderately sensitive activity (10 mm). As depicted in the same table, *A. baumannii* (12.5 to 13.5 mm) and *P. aeruginosa* (10.06 to 12.5 mm) were deemed as moderately sensitive to all origins of Eos. Based on the zone inhibition (8 to 9.5 mm), the *E. coli* was regarded as slightly sensitive to the volatile compounds of the four countries. The comparison of the inhibition activity according to the origin was not significant (*p* > 0.05), while the comparison between all strains tested is very highly significant (*p* < 0.001).

The MICs were measured using the microdilution method on 96-well plates, and the results obtained were recorded in Table 3.

All results reflect that the Eos from the different origins present an important inhibitory activity on all tested bacteria. The *MRSA* was the most sensitive bacterial strain to all Eos. The MICs recorded for the four origins were ranging from 3 to 10 *μ*l/ml with a slight difference with those observed in MBC values.

Regarding *A. baumannii* and *E. coli*, all MICs values were equal to those of MBCs (10 to 20 *μ*l/ml). As for *P. aeruginosa,* there was a difference between the MIC of India (10 *μ*l/ml) and MICs of Syria, Saudi Arabia, and Morocco (20 *μ*l/ml), but the MBCs values were the same for India, Morocco, and Saudi Arabia (30 *μ*l/ml) with an increase in the MBC of Syria Eo (40 *μ*l/ml).

## 4. Discussion

Regarding the chemical profile, the results obtained in our study displayed a different chemical profile with the literature when compared with the *NS* Eos from Iran, Tunisia, and Algeria [18–20], which confirms that the composition of the Eos is highly influenced by climatic conditions and also by the difference of the geographical location as mentioned in [21].

It is widely known that the volatile compounds are endowed with a great capacity to interrupt the production of energy on the mitochondrial membrane by damaging the proton pump; also, these compounds have the ability to inhibit the synthesis of structural macromolecules and growth regulators [22,23].

The antibacterial activity of *NS* volatile compounds for the studied countries (India, Saudi Arabia, Morocco, and Syria) could be associated to the different molecules identified with the GC-MS such as *α*-phellandrene, *β*-cymene, *β*-pinene, and thymol.

The obtained results indicate that the Eos originated from the four countries studied exert an antibacterial activity that was more potent in Gram-positive than the Gram-negative bacteria, and this could be due to the presence of an intrinsic system of resistance to Eos. Man et al. [24] indicated that the reaction of bacteria depends mainly on their morphology (bacterial wall), the peptidoglycan present on the Gram-positive bacterial wall allows the hydrophobic molecules to reach the internal environment, while the lipopolysaccharide present on the Gram-negative bacteria enables the passage of small hydrophilic molecules which are not affected by the resistance, and this is due to the abundance of porin proteins [25].

Concerning the antibacterial effect, it was reported that Tunisian *NS* Eos have shown an antibacterial activity against two strains the *E.coli* (IC_50_ 62.0 *μ*g/ml) and *S. aureus* (IC_50_ 12.0 *μ*g/ml); also, they suggested that these effects could be attributed to thymoquinone and longifolene [18].

In another study, the Eos showed an important activity on different multiresistant Gram-positive and Gram-negative strains, but no effect was observed on *A. baumannii,* which is contradictory to our results where all Eos studied have shown an inhibition of bacteria with an MIC value ranging from 10 to 20 *μ*l/ml [26], while in Deloer and Bari's study [27], the Eo had an important activity with low concentration on various strains with the exception of *E. coli* where the Eos were not active in all tested concentrations which was different from our findings where the Eos were active on *E. coli* with a MIC (20 *μ*l/ml). The Eos in Singh et al.'s [28] study showed an effect on *E. coli*, which correlates with the results registrated in our study.

Işcan et al.'s [29] study has revealed that *α*-phellandrene, the major compound in the Eos belonging to Saudia Arabia, Morocco, and Syria (20.03–30.54%), has a weak antibacterial activity toward several bacterial strains such as *S. aureus* (MRSA), *E. coli*, and *P. aeruginosa* at a concentration that varies from 1 to > 4 mg/ml. It was also mentioned that *α*-phellandrene could contribute to the antibacterial effect [30]. The *β*-pinene present in the Eos of the different countries studied with a percentage ranging from 4.53–9.77% and *α*-pinene present in Moroccan (6.29%) and Syrian (4.07%) Eos have showed an important inhibitory activity of *S. aureus* with an MIC value equal to 20 *μ*l/ml [31].

The research of Saad and Muller [23] shows that the hydroxyl group present on the thymol is responsible for the antimicrobial activity. Mosolygó et al. [32] have shown that the thymol, a monoterpene present in the essential oil of India (4.64%), Saudi Arabia (8.39%), and Morocco (7%), exhibits an antibacterial activity on both *S. aureus* and *E. coli*, and the MIC obtained was, respectively, 0.31 and 5 mg/ml. It was also reported that the thymol causes widening of the cell membrane which leads to a passive diffusion of ions between the spread phospholipids toward the external area [3,33].

The *β*-cymene, a carvacrol precursor of one of the major compounds present in all samples tested with a percentage between 12.31 and 23.82%, has an important affinity to the liposomal membrane which causes its extension [34], and it was also found that cymene has no significant effect on protein synthesis in *E. coli* while a significant effect was observed on motility decreasing [35].

## 5. Conclusions

The chemical compounds identified in this work using GC-MS were obtained by hydrodistillation followed by a study of the chemical variation and an evaluation of their antibacterial effect. Despite the geographical distribution of *NS* seeds, a great similarity was observed in oil composition and also on the activity exhibited on multidrug-resistant bacteria. Gram-positive organisms were generally more sensitive to Eos than gram-negative organisms. Thus, *NS* Eos provide a rich source of phytochemical compounds that could be used in the treatment of different infectious diseases caused by microbial agents.

## Figures and Tables

**Table 1:** Chemical constituents identified in the *Nigella sativa* L. essential oils of five countries.

Compounds	Retention time	India	Saudi Arabia	Morocco	Syria	France
*α*-Phellandrene	4.97 ± 0.00	10.58 ± 0.50	30.54 ± 1.01	29.64 ± 0.19	20.03 ± 0.56	4.31 ± 0.01
*α*-Pinene	5.10 ± 0.00			6.29 ± 0.04	4.07 ± 0.06	1.07 ± 0.01
UC	5.27 ± 0.00	0.12 ± 0.01	0.06 ± 0.01	0.13 ± 0.00	0.14 ± 0.03	
Camphene	5.36 ± 0.00	0.15 ± 0.02	0.15 ± 0.01	0.12 ± 0.03	0.18 ± 0.04	0.19 ± 0.03
UC	5.44 ± 0.00	0.05 ± 0.02	±	0.11 ± 0.01	0.11 ± 0.06	0.20 ± 0.06
UC	5.67 ± 0.00					1.11 ± 0.04
*β*-Phellandrene	5.74 ± 0.00	4.16 ± 0.07	4.42 ± 0.12	2.87 ± 0.00	2.39 ± 0.03	0.13 ± 0.00
*β*-Pinene	5.82 ± 0.00	8.59 ± 0.10	9.77 ± 0.33	6.22 ± 0.01	4.53 ± 0.07	0.54 ± 0.04
*β*-Myrcene	5.98 ± 0.00	0.17 ± 0.03	0.08 ± 0.02	0.15 ± 0.02	0.42 ± 0.08	0.42 ± 0.00
Ocimene	6.02 ± 0.00					0.31 ± 0.00
UC	6.04 ± 0.01	0.05 ± 0.01			0.33 ± 0.01	
Pseudocumol	6.07 ± 0.00	0.11 ± 0.01	0.01 ± 0.00	0.32 ± 0.01	0.21 ± 0.01	0.50 ± 0.01
UC	6.18 ± 0.00	0.07 ± 0.04	0.02 ± 0.01	0.05 ± 0.00	0.26 ± 0.01	0.21 ± 0.00
*α*-Thujene	6.24 ± 0.00	0.14 ± 0.03	0.07 ± 0.03	0.11 ± 0.03		
(+)-4-Carene	6.26 ± 0.00				1.28 ± 0.11	
UC	6.35 ± 0.00	0.10 ± 0.03	0.06 ± 0.02	0.20 ± 0.04		
D-Limonene	6.39 ± 0.00					14.63 ± 1.42
*α*-Terpinen	6.44 ± 0.00	1.67 ± 0.09	3.34 ± 0.17	2.25 ± 0.00		
UC	6.45 ± 0.00					1.41 ± 0.29
UC	6.51 ± 0.00	0.06 ± 0.01	0.07 ± 0.01	±		±
*β*-Cymene	6.56 ± 0.01	14.22 ± 0.08	12.31 ± 0.67	23.82 ± 0.04	14.61 ± 1.27	0.29 ± 0.00
UC	6.60 ± 0.00				0.27 ± 0.02	0.19 ± 0.01
UC	6.71 ± 0.00				0.23 ± 0.01	
UC	6.77 ± 0.00				0.39 ± 0.05	
Thujol	6.98 ± 0.00	0.05 ± 0.00	0.02 ± 0.00	0.02 ± 0.01	0.73 ± 0.10	0.63 ± 0.12
ɤ-Terpinene	7.14 ± 0.00	6.38 ± 0.34	1.89 ± 0.10	1.25 ± 0.06	5.77 ± 0.11	0.18 ± 0.07
UC	7.24 ± 0.00				0.44 ± 0.06	
cis-*β*-Terpineol	7.29 ± 0.00	0.18 ± 0.01	0.17 ± 0.02	0.11 ± 0.01	0.48 ± 0.08	0.96 ± 0.12
Terpinolen	7.64 ± 0.00	0.37 ± 0.02	0.25 ± 0.03	0.15 ± 0.02	63 ± 0.08	0.58 ± 0.13
UC	7.68 ± 0.00				0.25 ± 0.02	
3-Caranol	7.76 ± 0.00				0.28 ± 0.04	0.20 ± 0.06
Neodihydrocarveol	7.77 ± 0.00	3.34 ± 0.19	2.51 ± 0.03	1.63 ± 0.00	3.50 ± 0.25	0.52 ± 0.01
UC	8.04 ± 0.00	0.04 ± 0.01	0.04 ± 0.01	0.04 ± 0.01	0.31 ± 0.00	
4-Caranol	8.14 ± 0.00	18.81 ± 0.57	14.27 ± 0.11	9.77 ± 0.08	9.85 ± 0.65	2.05 ± 0.03
UC	8.29 ± 0.00	0.07 ± 0.03	0.05 ± 0.00	0.03 ± 0.01	0.35 ± 0.03	0.49 ± 0.01
UC	8.45 ± 0.00	0.27 ± 0.01	0.43 ± 0.04	0.25 ± 0.03	0.46 ± 0.00	0.22 ± 0.03
UC	8.57 ± 0.00	0.04 ± 0.00	0.02 ± 0.00	0.01 ± 0.01	0.36 ± 0.07	0.47 ± 0.03
Limonene oxide	8.57 ± 0.00				0.66 ± 0.09	0.29 ± 0.07
Camphor	8.62 ± 0.00	0.08 ± 0.01	0.07 ± 0.02	0.05 ± 0.00	0.27 ± 0.04	0.96 ± 0.14
*β*-Cyclocitral	8.75 ± 0.00	0.12 ± 0.02	0.05 ± 0.01	0.03 ± 0.00	0.17 ± 0.01	0.24 ± 0.10
UC	8.77 ± 0.00					0.94 ± 0.18
p-Cymen-8-ol	8.87 ± 0.00				0.24 ± 0.02	0.18 ± 0.01
Limonene epoxide	8.89 ± 0.00	1.02 ± 0.07	0.78 ± 0.01	0.52 ± 0.01	0.42 ± 0.04	0.45 ± 0.07
UC	8.99 ± 0.00	0.11 ± 0.00	0.04 ± 0.01	0.03 ± 0.01	0.40 ± 0.02	0.16 ± 0.02
4-Carvomenthenol	9.10 ± 0.00	3.35 ± 0.03	2.11 ± 0.01	1.44 ± 0.06	1.67 ± 0.03	0.47 ± 0.13
UC	9.19 ± 0.00	0.24 ± 0.02	0.20 ± 0.00	0.15 ± 0.01	0.12 ± 0.03	
UC	9.25 ± 0.00	0.03 ± 0.00	0.02 ± 0.00	0.01 ± 0.00	0.32 ± 0.07	0.82 ± 0.04
UC	9.30 ± 0.00	0.11 ± 0.01	0.04 ± 0.00	0.03 ± 0.00	0.33 ± 0.03	
UC	9.40 ± 0.00	0.10 ± 0.01	0.08 ± 0.03	0.07 ± 0.01		0.39 ± 0.02
*β*-Cyclocitral	9.50 ± 0.00	2.10 ± 0.13	2.0 ± 0.05	1.39 ± 0.02	1.06 ± 0.01	0.24 ± 0.01
UC	9.54 ± 0.00				0.19 ± 0.01	0.24 ± 0.07
UC	9.55 ± 0.00	0.04 ± 0.00		0.02 ± 0.01	0.46 ± 0.02	0.22 ± 0.05
UC	9.74 ± 0.00	0.34 ± 0.04	0.13 ± 0.00	0.08 ± 0.00	0.35 ± 0.02	0.15 ± 0.01
Solanone	9.98 ± 0.00				0.42 ± 0.02	0.51 ± 0.03
Cuminal	10.08 ± 0.00	0.09 ± 0.01	0.24 ± 0.06	0.13 ± 0.02	0.19 ± 0.02	0.51 ± 0.03
Carvone	10.14 ± 0.00	0.18 ± 0.01	0.24 ± 0.01	0.010.15 ± 0.04	0.23 ± 0.01	1.01 ± 0.12
cis-Verbenol	10.19 ± 0.00				0.17 ± 0.03	0.40 ± 0.00
Thymoquinon	10.21 ± 0.00	0.32 ± 0.03	0.70 ± 0.10	0.50 ± 0.06	0.82 ± 0.11	
UC	10.25 ± 0.00				0.08 ± 0.01	
cis-Verbenol	10.27 ± 0.00	0.12 ± 0.01	0.08 ± 0.00	0.05 ± 0.01	0.19 ± 0.00	0.96 ± 0.01
UC	10.31 ± 0.00	0.02 ± 0.01				0.63 ± 0.14
Estragole	10.32 ± 0.00					20.22 ± 0.59
4-Carene	10.38 ± 0.00	0.24 ± 0.02	0.13 ± 0.01	0.09 ± 0.01	0.33 ± 0.08	0.64 ± 0.04
Carvacrol	10.49 ± 0.00				2.30 ± 0.09	1.22 ± 0.07
UC	10.71 ± 0.00				0.18 ± 0.04	0.28 ± 0.04
Bornyl acetate	10.76 ± 0.00	1.14 ± 0.09	0.84 ± 0.05	0.59 ± 0.05	1.18 ± 0.04	
UC	10.86 ± 0.00	0.09 ± 0.01	0.03 ± 0.00	±	0.12 ± 0.01	
Thymol	10.92 ± 0.00	4.64 ± 0.01	8.39 ± 0.39	7.00 ± 0.03	±	
UC	11.14 ± 0.00	0.07 ± 0.03	0.02 ± 0.00	0.04 ± 0.00	0.18 ± 0.03	
UC	11.30 ± 0.00	0.04 ± 0.01	0.02 ± 0.01			
UC	11.55 ± 0.00				0.20 ± 0.01	
Cedrene	11.60 ± 0.00					0.22 ± 0.01
*β*-Terpinyl acetate	11.65 ± 0.00	0.10 ± 0.03	0.02 ± 0.00	0.18 ± 0.02		±
*α*-Longipinene	11.78 ± 0.00	2.23 ± 0.09	0.29 ± 0.02	0.18 ± 0.01	1.22 ± 0.05	0.51 ± 0.10
UC	12.05 ± 0.00	0.06 ± 0.02			0.15 ± 0.03	
Patchoulene	12.07 ± 0.00				4.00 ± 0.05	3.29 ± 0.00
Columbin	12.11 ± 0.00	0.14 ± 0.02	0.05 ± 0.01	0.02 ± 0.00		
UC	12.16 ± 0.00	0.04 ± 0.01	0.02 ± 0.00		0.12 ± 0.04	0.42 ± 0.00
Aromadendrene	12.22 ± 0.00				0.29 ± 0.04	0.69 ± 0.10
UC	12.31 ± 0.00	0.04 ± 0.01	0.02 ± 0.01	0.01 ± 0.00	0.18 ± 0.01	0.19 ± 0.05
UC	12.41 ± 0.00	0.06 ± 0.01	0.02 ± 0.00	0.04 ± 0.02	0.18 ± 0.00	0.24 ± 0.01
UC	12.51 ± 0.00	0.06 ± 0.02				
Longicyclene	12.56 ± 0.00	0.31 ± 0.02	0.02 ± 0.00	±	0.00	
*β*-Chamigrene	12.62 ± 0.00	9.54 ± 0.49	1.54 ± 0.08	1.14 ± 0.02		
UC	12.68 ± 0.00	0.02 ± 0.01			0.13 ± 0.03	0.19 ± 0.03
*α*-Bisabolene	12.77 ± 0.00	0.18 ± 0.02	0.06 ± 0.01	0.03 ± 0.01	0.21 ± 0.04	0.62 ± 0.00
Longifolene-(V4)	12.91 ± 0.00					2.61 ± 0.02
UC	12.99 ± 0.00					1.20 ± 0.07
UC	13.00 ± 0.00	0.05 ± 0.01	0.04 ± 0.02	0.01 ± 0.00	0.25 ± 0.02	0.20 ± 0.09
Methyl undecyl ketone	13.07 ± 0.00				1.51 ± 0.11	0.79 ± 0.04
UC	13.12 ± 0.01	0.03 ± 0.01	0.04 ± 0.01	0.01 ± 0.00	0.75 ± 0.09	
UC	13.17 ± 0.00				0.87 ± 0.07	0.47 ± 0.07
a-Curcumene	13.28 ± 0.00	0.11 ± 0.03	0.03 ± 0.00	0.02 ± 0.00		0.25 ± 0.02
b-Sesquiphellandrene	13.38 ± 0.00					0.36 ± 0.01
UC	13.49 ± 0.00	0.06 ± 0.01	0.05 ± 0.01	0.03 ± 0.02	0.32 ± 0.08	
2-Tridecanone	13.57 ± 0.00	0.38 ± 0.02	0.11 ± 0.02	0.07 ± 0.02	1.13 ± 0.03	1.40 ± 0.14
UC	13.64 ± 0.00	0.07 ± 0.02	0.04 ± 0.01	0.02 ± 0.01	0.19 ± 0.02	1.14 ± 0.04
*β*-Bisabolene	13.83 ± 0.00	0.17 ± 0.03	0.04 ± 0.01	0.03 ± 0.01	0.14 ± 0.08	0.15 ± 0.01
UC	13.99 ± 0.00	0.06 ± 0.01	0.04 ± 0.01	0.02 ± 0.00	±	0.23 ± 0.07
Isoledene	14.15 ± 0.00	0.15 ± 0.02	0.03 ± 0.01	0.01 ± 0.01	0.16 ± 0.01	0.37 ± 0.04
UC	14.40 ± 0.00	0.04 ± 0.00	0.04 ± 0.01	0.02 ± 0.01	0.15 ± 0.04	0.92 ± 0.13
UC	14.72 ± 0.00	0.04 ± 0.01		0.02 ± 0.00	0.13 ± 0.03	0.79 ± 0.06
a-Pentylcinnamaldehyde	14.78 ± 0.00					0.47 ± 0.08
UC	14.85 ± 0.00	0.28 ± 0.03	0.13 ± 0.03	0.07 ± 0.02	0.15 ± 0.06	1.21 ± 0.03
Ar-tumerone	15.05 ± 0.00	±	±	±	±	0.55 ± 0.04
UC	15.06 ± 0.00	0.22 ± 0.03	0.10 ± 0.01	0.06 ± 0.00	0.24 ± 0.02	0.38 ± 0.10
UC	15.17 ± 0.00	0.07 ± 0.01	0.02 ± 0.01	0.05 ± 0.05	0.18 ± 0.06	1.30 ± 0.14
Curlone	15.41 ± 0.00					0.67 ± 0.06
UC	15.62 ± 0.00	0.09 ± 0.02	0.05 ± 0.01	0.01 ± 0.00	0.21 ± 0.10	2.38 ± 0.01
UC	15.87 ± 0.00	0.15 ± 0.03	0.06 ± 0.02	0.05 ± 0.02		1.09 ± 0.04
UC	16.04 ± 0.00	0.09 ± 0.02		0.02 ± 0.01		
UC	16.09 ± 0.00	0.11 ± 0.02	0.05 ± 0.01			
Globulol	16.15 ± 0.00	0.34 ± 0.04	0.05 ± 0.00	0.03 ± 0.01	0.13 ± 0.04	1.42 ± 0.00
Palmitic acid	16.55 ± 0.00					0.16 ± 0.01
UC	16.64 ± 0.00	0.21 ± .05	0.15 ± 0.03	0.06 ± 0.02	0.27 ± 0.03	2.09 ± 0.00
UC	16.88 ± 0.00	0.09 ± 0.02	0.03 ± 0.01	0.02 ± 0.00	0.23 ± 0.08	
Biformene	18.18 ± 0.02					1.07 ± 0.05
UC	18.24 ± 0.00					4.16 ± 0.17
UC	18.34 ± 0.01					1.85 ± 0.06
7,10-Octadecadienoic acid	19.21 ± 0.01					0.55 ± 0.04
UC	19.39 ± 0.00					2.97 ± 0.03

**Table 2:** Zone of inhibition means (mm) of *Nigella sativa* essential oils (Eos) for tested bacterial strains.

Origins	Bacterial strains			
	*MRSA*	*A. baumannii*	*E. coli*	*P. aeruginosa*
India	15,1 ± 0,13	12,5 ± 0	8 ± 0,28	12,03 ± 0,11
Saudi Arabia	10 ± 0,25	13,5 ± 0,22	8,5 ± 0,09	11,5 ± 0,21
Morocco	14 ± 0,33	13 ± 0,02	9,5 ± 0,33	12,5 ± 0,16
Syria	15 ± 0,67	13 ± 0,27	9 ± 0,13	10,06 ± 0,09

**Table 3:** The MIC and MBC in *μ*l/ml of *Nigella sativa* essential oils against tested bacteria.

Origins	Bacterial strains							
	*MRSA*		*A. baumannii*		*E. coli*		*P. aeruginosa*	
	MIC	MBC	MIC	MBC	MIC	MBC	MIC	MBC
India	10	10	20	20	20	20	10	30
Saudi Arabia	3	5	20	20	20	20	20	30
Morocco	5	10	20	20	20	20	20	30
Syria	3	3	10	10	20	20	20	40

MIC and MBC are minimal inhibitory concentration and minimal bactericidal concentration, respectively.

## Data Availability

All data used to support the finding of our study are available online and from the corresponding author upon request.
